# Sparing effects of selenium and ascorbic acid on vitamin C and E in guinea pig tissues

**DOI:** 10.1186/1475-2891-6-7

**Published:** 2007-03-26

**Authors:** Jesse Bertinato, Nick Hidiroglou, Robert Peace, Kevin A Cockell, Keith D Trick, Penny Jee, Alex Giroux, Réné Madère, Giuseppe Bonacci, Monica Iskandar, Stephen Hayward, Nicholas Giles, Mary R L'Abbé

**Affiliations:** 1 Nutrition Research Division, Food Directorate, Health Products and Food Branch, Health Canada, Sir Frederick G. Banting Research Centre, Ottawa, ON, Canada; 2 Bureau of Biostatistics and Computer Applications, Food Directorate, Health Products and Food Branch, Health Canada, Sir Frederick G. Banting Research Centre, Ottawa, ON, Canada

## Abstract

**Background:**

Selenium (Se), vitamin C and vitamin E function as antioxidants within the body. In this study, we investigated the effects of reduced dietary Se and L-ascorbic acid (AA) on vitamin C and α-tocopherol (AT) status in guinea pig tissues.

**Methods:**

Male Hartley guinea pigs were orally dosed with a marginal amount of AA and fed a diet deficient (Se-D/MC), marginal (Se-M/MC) or normal (Se-N/MC) in Se. An additional diet group (Se-N/NC) was fed normal Se and dosed with a normal amount of AA. Guinea pigs were killed after 5 or 12 weeks on the experimental diets at 24 and 48 hours post AA dosing.

**Results:**

Liver Se-dependent glutathione peroxidase activity was decreased (*P *< 0.05) in guinea pigs fed Se or AA restricted diets. Plasma total glutathione concentrations were unaffected (*P *> 0.05) by reduction in dietary Se or AA. All tissues examined showed a decrease (*P *< 0.05) in AA content in Se-N/MC compared to Se-N/NC guinea pigs. Kidney, testis, muscle and spleen showed a decreasing trend (*P *< 0.05) in AA content with decreasing Se in the diet. Dehydroascorbic acid concentrations were decreased (*P *< 0.05) in several tissues with reduction in dietary Se (heart and spleen) or AA (liver, heart, kidney, muscle and spleen). At week 12, combined dietary restriction of Se and AA decreased AT concentrations in most tissues. In addition, restriction of Se (liver, heart and spleen) and AA (liver, kidney and spleen) separately also reduced AT in tissues.

**Conclusion:**

Together, these data demonstrate sparing effects of Se and AA on vitamin C and AT in guinea pig tissues.

## Background

Vitamin C is a water soluble antioxidant. In contrast to many mammals, humans (and guinea pigs) are unable to synthesise vitamin C due to the lack of the enzyme L-gulono-gamma-lactone oxidase [1] and therefore must rely on diet for maintaining adequate levels of the vitamin. In tissues, the active form of vitamin C, L-ascorbic acid (AA), can be regenerated by the reduction of its oxidised forms, dehydroascorbic acid (DHAA) and the ascorbate free radical in a process mediated by glutathione (GSH) [2-6]. Notably, however, other systems have also been implicated in the regeneration of AA [7,8].

Selenium (Se) and vitamin E also function as important antioxidants within the body. Se is an essential trace element that functions in oxidant defence as a component of selenoproteins [9,10]. Vitamin E is a lipid soluble antioxidant present in cell membranes where it plays a vital role in protecting against lipid peroxidation [11-13]. Vitamin E refers to several structurally related compounds; however, α-tocopherol (AT) is the predominant form found in animal tissues. Like vitamin C, vitamin E must be obtained from the diet.

The importance of maintaining adequate levels of Se, vitamin C and vitamin E is underscored by studies indicating that low antioxidant status may be associated with increased risk of developing various diseases [14-16]. Se has been shown to spare both AA [7,8] and AT [17]. Further, sparing effects of AA on AT have also been reported [18-20]. Given that Se, vitamin C and vitamin E activities are interconnected, it is important to understand how deficiency in one or two of these antioxidants influences the other(s). In this study we sought to explore the sparing effects of Se and AA on vitamin C and AT in guinea pig, an *in vivo *model that cannot synthesise vitamin C.

## Methods

### Animals and test diets

On arrival, male Hartley guinea pigs (~ 10 days old) (Elm Hill Breeding Labs, Inc., Chelmsford, MA) were subjected to a 2 week adaptation period. Following the adaptation period, guinea pigs (n = 22/diet group) had free access to one of 4 test diets (Table 1) and demineralised drinking water. Normal or marginal amounts of AA were given to each guinea pig in a 0.5 mL aqueous solution via oral dosing by gavage three times per week (i.e. Monday, Wednesday and Friday). Amount of AA was calculated from the previous day's mean body weight for the diet group [2.4 (normal) or 0.3 (marginal) mg AA/100 g body weight]. Normal and marginal AA levels were chosen based on the AA requirement for growing guinea pigs and previous studies demonstrating suboptimal dietary AA levels [21-23]. Test diets were torula yeast-based diets deficient in Se and similar to diets previously used to induce Se deficiency in guinea pigs [24]. Test diets were supplemented with 0 (deficient), 0.05 (marginal) or 0.20 (normal) mg Se/kg diet.

**Table 1:** Composition of experimental diets

Ingredients	Diets			
	
	Se-D/MC	Se-M/MC	Se-N/MC	Se-N/NC
	*g/kg diet*			
Torula Yeast	400	400	400	400
Non-nutritive fibre	150	150	150	150
Cornstarch	142	142	142	142
Mineral mix^1^	90	90	90	90
Sucrose	73.1	73.1	73.1	73.1
Stripped corn oil^2^	73	73	73	73
Dextrinised starch	51.9	51.9	51.9	51.9
Vitamin mix^3^	10	10	10	10
Choline chloride	4	4	4	4
L-methionine	3	3	3	3
L-arginine	3	3	3	3
DL-α-tocopherol acetate^4^	40	40	40	40
Se^5^	0	0.05	0.20	0.20
L-ascorbic acid^6^	0.3	0.3	0.3	2.4

^1 ^Dyets no 200151 salt mix for Reid-Briggs guinea pig diet.

^2 ^Containing 0.2 mg *Tert*-butylhydroquinone/g stripped corn oil.

^3 ^Dyets no 300151 vitamin mix for Reid-Briggs guinea pig diet (Vitamin E omitted).

^4 ^IU/kg diet; added via stripped corn oil.

^5 ^mg/kg diet; added as Na_2_SeO_4_.

^6 ^mg L-ascorbic acid/100 g body weight.

Guinea pigs were killed following an overnight fast by exsanguination while anesthetised with 3% isoflurane. Half the guinea pigs per diet group were killed after 5 weeks and the remainder after 12 weeks on the experimental diets at 24 and 48 hrs post AA dosing. Blood was withdrawn from the abdominal aorta and collected in heparinised tubes. Plasma was separated from cells by centrifugation (1000 × *g*, 20 min, 4°C). Skeletal muscle (from quadriceps) and soft tissues were extracted and immediately frozen in liquid nitrogen. Plasma and tissues were stored at -80°C until analysis. The Health Canada Animal Care Committee approved the experimental protocol. Guinea pigs were treated in accordance with the guidelines of the Canadian Council on Animal Care.

### Determination of vitamin C and α-tocopherol in tissues

AA and total vitamin C (following reduction of the sample with homocysteine) were measured by reverse-phase HPLC with electrochemical detection as described [25]. DHAA was calculated as the difference between total vitamin C and AA. Excised tissues were immediately frozen in liquid nitrogen, a procedure that has been shown to prevent oxidation of vitamin C [26]. To further prevent oxidation of vitamin C, plasma and tissue homogenates were preserved by treating with metaphosphoric acid to a final concentration of 0.85% w/v. AA and DHAA standards could be completely recovered when spiked into tissue homogenates indicating that with this method both AA and DHAA are stable in a tissue matrix [27]. Further, time course experiments revealed that AA and DHAA concentrations remained constant over the course of 90 minutes indicating that AA was not being converted to DHAA. AA concentrations determined using this method were also closely correlated with AA concentrations determined using the 2, 4-dinitrophenylhydrazine method [28]. AT content was determined by reverse-phase HPLC with fluorescence detection [29].

### Enzyme and other assays

Se-dependent glutathione peroxidase (Se-GSHPx) activity was measured essentially as described [30] using a SPECTRAmax PLUS microplate spectrophotometer (Molecular Devices, Sunnyvale, CA). Liver extracts were prepared by homogenising in 0.2% Triton-X-100. Se-GSHPx activity is expressed as U/g protein, where one unit of activity catalyses the oxidation of 1.0 mmol of reduced NADPH/minute. Total plasma GSH was determined by HPLC using a manual adaptation of the automated NaBH_4 _reduction and monobromobimane derivatization procedures described previously [31,32]. Liver cytosolic and plasma protein carbonyls were determined by slot-blot immunoassay using reagents from an OxyBlot Protein Oxidation Detection Kit (Intergen, NY, USA) as previously described [33]. Lipid peroxide concentrations in liver homogenates and plasma were determined using a commercially available kit (LPO-CC Lipid Peroxides, Kamiya Biomedical, Seattle, WA, USA). Protein concentration was determined by the bicinchoninic acid method [34].

### Statistical analyses

Data were analysed by one-way ANOVA and differences between means were determined by Fisher's least significant difference test. For tissue vitamin C, data were analysed using univariate ANOVA with diet as the main effect. Since the variability of tissue AA levels increased with an increase in mean, AA data were transformed using the square root transformation. Two contrasts were included in the analyses to test for an effect of Se or AA intake on tissue AA, DHAA and total vitamin C concentrations. The Se-D/MC, Se-M/MC and Se-N/MC diet groups were used to test for an effect of Se intake. These diet groups were also used to determine whether a decreasing or increasing trend was present as the amount of Se decreased in the diet. Trend here refers to an overall increasing or decreasing response to a decrease in dose. To test for an effect of AA intake, the Se-N/MC and Se-N/NC diet groups were compared. For vitamin C analyses, data from guinea pigs killed at week 5, 48 hrs post AA dosing and week 12, 24 hrs post AA dosing were combined in order to increase the power of the statistical comparisons. The ability to combine these data is predicated on the similarity of the response (i.e. tissue vitamin C concentrations) of week 5, 48 hrs and week 12, 24 hrs guinea pigs to changes in dietary Se or AA. Similarity of the response was determined by ANOVA. Data are shown as means ± SEM. Statistical significance was set at *P *< 0.05. Data were analysed using Statistica 7 (StatSoft, Tulsa, OK) and SAS (SAS Canada, Ottawa, Canada) software.

## Results

To investigate the sparing effects of Se on vitamin C and AT when intake of AA is low, guinea pigs were orally dosed with a marginal amount of AA and fed a diet deficient (Se-D/MC), marginal (Se-M/MC) or normal (Se-N/MC) in Se. An additional diet group (Se-N/NC) dosed with a normal amount of AA and fed a normal Se diet was included in the experimental protocol to allow investigation of the sparing effects of AA on vitamin C and AT (comparison with diet group Se-N/MC). Approximately one third of the guinea pigs fed the Se-D/MC diet developed paralysis of their hind limbs and showed poor mobility beginning as early as 4 weeks on the diet. Three Se-D/MC guinea pigs died or were euthanised prior to the week 5 necropsy and two prior to the week 12 necropsy.

At week 5, liver Se-GSHPx activity showed a dose-dependent decrease (*P *< 0.05) with decreasing amounts of Se in the diet, confirming induction of graded levels of Se status in the guinea pigs (Table 2). At week 12, Se-D/MC guinea pigs had lower Se-GSHPx activity compared to Se-M/MC or Se-N/MC guinea pigs. Se-N/MC guinea pigs had lower Se-GSHPx activity compared to Se-N/NC guinea pigs at week 12. Plasma total GSH concentrations were similar (*P *> 0.05) in guinea pigs fed the different test diets, consistent with a previous study showing no change in GSH levels with decreased Se and vitamin C status [7]. However, plasma GSH concentrations were lower (*P *< 0.05) in week 12 compared to week 5 guinea pigs for all diet groups.

**Table 2:** Liver Se-GSHPx activity and plasma GSH concentration of guinea pigs after 5 and 12 weeks on the experimental diets^1^

Diet Group	Liver Se-GSHPx (U/g protein)		Plasma GSH (uM/L)	
	
	Week 5	Week 12	Week 5	Week 12
Se-D/MC	15.02 ± 1.32^a^	14.36 ± 1.49^a^	7.55 ± 0.45^a^	4.23 ± 0.20^a^
	(n = 8)	(n = 9)	(n = 8)	(n = 7)
Se-M/MC	33.97 ± 1.87^b^	30.84 ± 1.58^b^	8.44 ± 0.85^a^	3.86 ± 0.13^a^
	(n = 11)	(n = 11)	(n = 8)	(n = 8)
Se-N/MC	46.78 ± 2.70^c^	35.52 ± 1.90^b^	7.38 ± 0.49^a^	4.12 ± 0.31^a^
	(n = 11)	(n = 10)	(n = 8)	(n = 8)
Se-N/NC	57.04 ± 6.46^c^	43.12 ± 1.70^c^	7.52 ± 0.92^a^	4.33 ± 0.38^a^
	(n = 11)	(n = 11)	(n = 8)	(n = 9)

^1 ^Data from guinea pigs killed at both 24 and 48 hrs post AA dosing.

^2 ^Values in a column without a common letter differ, *P *< 0.05. Values are means ± SEM.

^3 ^n values are in parentheses.

**Figure 1:**
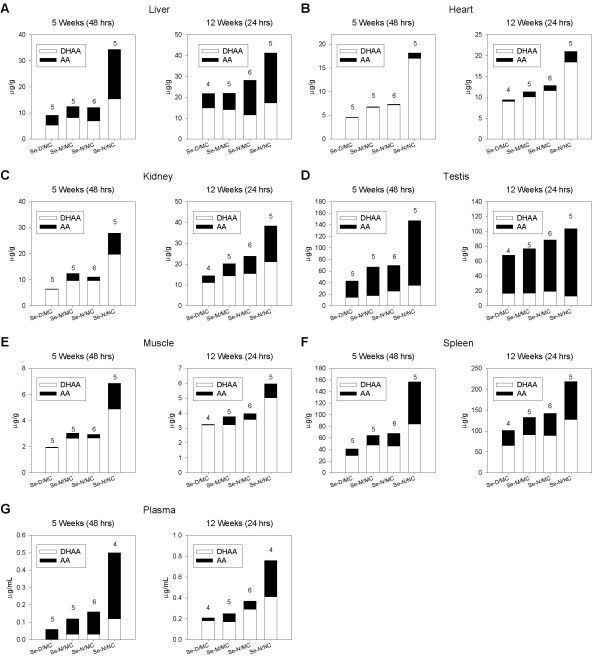
**Vitamin C concentrations in tissues of guinea pigs fed the experimental diets**. Bars signify the amount of total vitamin C (DHAA + AA) in tissues of guinea pigs fed the Se-D/MC, Se-M/MC, Se-N/MC or Se-N/NC diets. The proportions of DHAA (white portion of bar) and AA (black portion of bar) are shown. Values are reported as the mean. Number of tissues analysed for each diet group are indicated above the bars. For each tissue, data are shown for guinea pigs killed after 5 and 12 weeks on the experimental diets at 48 and 24 hrs post AA dosing, respectively.

Tissue vitamin C and AT concentrations are only presented for guinea pigs killed at week 5, 48 hrs post AA dosing and week 12, 24 hrs post dosing (see Discussion). Proportion of DHAA to AA in each tissue was similar between week 5 and 12 guinea pigs (Fig. 1). The only exception was plasma, where most of the vitamin C was present as AA at week 5 and as DHAA at week 12 (Fig. 1G). The ratio of DHAA to AA varied markedly between tissues. In testis, the majority of vitamin C was AA (Fig. 1D), whereas in heart and muscle most of the vitamin C was DHAA (Fig. 1B and 1E).

Because of the large variability in vitamin C concentrations between individual guinea pigs and the relatively small number of guinea pigs analysed per diet group, vitamin C data were combined for week 5, 48 hrs and week 12, 24 hrs guinea pigs to increase the power of the statistical comparisons (see Methods and Discussion). Effects of Se or AA intakes on tissue AA, DHAA and total vitamin C concentrations were determined by univariate ANOVA (Table 3). Differences between the Se-D/MC, Se-M/MC and Se-N/MC diet groups were determined to test for sparing effects of Se (Se Effect). Additional statistical analyses were performed to test whether there was a decreasing or increasing trend in vitamin C concentrations with decreasing Se in the diet [Se Effect (Trend)]. Differences between the Se-N/MC and Se-N/NC diet groups were determined to test for sparing effects of AA (AA Effect).

**Table 3:** Univariate significance tests for the effects of Se and AA intakes on vitamin C concentrations in guinea pig tissues^1^

Tissue	Se Effect^2^			AA Effect ^3^			Se Effect (Trend)^4^		
	
	AA	DHAA	Total	AA	DHAA	Total	AA	DHAA	Total
Liver	NSD	< 0.05	< 0.01	< 0.05	< 0.0001	< 0.0001	NSD	< 0.005	< 0.005
Heart	NSD	< 0.05	< 0.05	< 0.01	< 0.0001	< 0.0001	NSD	< 0.01	< 0.01
Kidney	< 0.05	NSD	NSD	< 0.005	< 0.0001	< 0.0001	< 0.05	NSD	NSD
Testis	NSD	NSD	< 0.05	< 0.0001	NSD	< 0.0001	< 0.05	NSD	< 0.005
Muscle	< 0.05	NSD	< 0.05	< 0.001	< 0.0001	< 0.0001	< 0.05	NSD	< 0.05
Spleen	NSD	< 0.05	< 0.01	< 0.0001	< 0.0001	< 0.0001	< 0.05	< 0.05	< 0.005
Plasma	NSD	NSD	NSD	< 0.0005^5^	NSD^5^	< 0.0001^5^	NSD	NSD	NSD

^1 ^Type III *P *values are shown; NSD = no statistical difference.

^2 ^Comparison between Se-D/MC, Se-M/MC and Se-N/MC guinea pigs killed at week 5 and 12, 48 and 24 hrs post AA dosing, respectively (n = 31).

^3 ^Comparison between Se-N/MC and Se-N/NC guinea pigs killed at week 5 and 12, 48 and 24 hrs post AA dosing, respectively (n = 22).

^4 ^Analyses to determine a decreasing or increasing trend with decreasing Se in the diet. Comparison of Se-D/MC, Se-M/MC and Se-N/MC guinea pigs killed at week 5 and 12, 48 and 24 hrs post AA dosing, respectively (n = 31). All significant values indicate a decreasing trend with decreasing Se in the diet, except for liver DHAA which indicates an increasing trend.

^5 ^n = 20.

Se intake affected (*P *< 0.05) AA concentrations in kidney and muscle (Table 3, Se Effect). Trend analyses confirmed that the differences detected reflected a decrease in AA concentrations with decreasing Se in the diet [Table 3, Se Effect (Trend)]. Significant differences in AA concentrations were not detected (*P *> 0.05) in liver, heart, testis, spleen and plasma. However, testis and spleen showed a decreasing trend for AA with reduction in dietary Se. Total vitamin C concentrations were affected by Se intake in liver, heart, testis, muscle and spleen and showed a decreasing trend with decreasing Se. A Se effect and similar decreasing trend was observed for DHAA in heart and spleen. In contrast, the Se effect on liver DHAA reflected an increasing trend with decreasing Se.

Se-N/MC guinea pigs had reduced (*P *< 0.05) AA concentrations in all tissues compared to Se-N/NC guinea pigs [Table 3, AA Effect]. DHAA concentrations in Se-N/MC guinea pigs were also reduced (*P *< 0.0001) in liver, heart, kidney, muscle and spleen. All tissues from Se-N/MC guinea pigs showed decreased (*P *< 0.0001) concentrations of total vitamin C.

At week 5, there were no significant (*P *> 0.05) differences in AT concentrations between diet groups for any of the tissues analysed (Table 4). At week 12, Se-D/MC guinea pigs had lower (*P *< 0.05) AT concentrations in liver, heart and spleen compared to Se-M/MC guinea pigs (Table 4). AT was lower in liver, kidney and spleen of Se-N/MC compared to Se-N/NC guinea pigs. In plasma, while sole restriction of Se or vitamin C showed no effects on AT concentrations, combined restriction of Se and vitamin C decreased AT (compare Se-D/MC and Se-N/NC). Combined restriction of Se and vitamin C also decreased AT in liver, heart, kidney and spleen. Collectively, these data indicate that reductions in dietary Se and AA singly or in combination decrease AT concentrations in guinea pig tissues.

**Table 4:** Effects of Se and AA intakes on α-tocopherol concentrations in guinea pig tissues^1^

Animals	Liver	Heart	Kidney	Testis	Muscle	Spleen	Plasma^2^
Week 5^3^	μg AT/g						
Se-D/MC (n = 5)	6.50 ± 1.57^a^	2.22 ± 0.67^a^	2.68 ± 0.88^a^	2.42 ± 0.65^a^	1.46 ± 0.36^a^	3.71 ± 0.86^a^	1.26 ± 0.28^a^
Se-M/MC (n = 5)	12.13 ± 3.42^a^	3.20 ± 0.86^a^	2.77 ± 0.81^a^	2.61 ± 0.84^a^	1.11 ± 0.42^a^	5.53 ± 1.22^a^	1.11 ± 0.18^a^
Se-N/MC (n = 6)	10.30 ± 2.88^a^	2.71 ± 1.12^a^	2.92 ± 1.11^a^	3.07 ± 0.92^a^	1.19 ± 0.49^a^	5.53 ± 1.23^a^	1.00 ± 0.31^a^
Se-N/NC (n = 5)	14.78 ± 4.11^a^	3.70 ± 1.41^a^	4.91 ± 1.15^a^	2.74 ± 0.63^a^	1.38 ± 0.34^a^	6.75 ± 1.63^a^	1.13 ± 0.33^a4^
Week 12^3^							
Se-D/MC (n = 4)	10.35 ± 3.58^a^	2.64 ± 0.77^a^	3.90 ± 0.92^a^	2.34 ± 0.59^a^	0.95 ± 0.32^a^	5.32 ± 1.58^a^	0.59 ± 0.27^a^
Se-M/MC (n = 5)	20.71 ± 1.54^bc^	6.79 ± 0.72^b^	5.46 ± 0.72^a^	2.98 ± 0.35^a^	1.68 ± 0.31^a^	8.75 ± 1.02^bc^	1.10 ± 0.15^ab^
Se-N/MC (n = 6)	15.10 ± 1.81^ab^	5.49 ± 0.67^ab^	5.12 ± 0.64^a^	2.49 ± 0.21^a^	1.53 ± 0.25^a^	7.37 ± 0.87^ab^	0.73 ± 0.11^ab^
Se-N/NC (n = 5)	23.87 ± 3.86^c^	6.37 ± 1.59^b^	7.98 ± 1.03^b^	3.63 ± 0.62^a^	1.66 ± 0.23^a^	10.76 ± 0.98^c^	1.48 ± 0.47^b^

^1 ^AT concentrations in tissues from guinea pigs killed at week 5 and 12, 48 and 24 hrs post AA dosing, respectively.

^2^μg AT/mL.

^3 ^For week 5 and week 12, values in a column without a common letter differ, *P *< 0.05. Values are means ± SEM.

^4 ^n = 4.

^5 ^n values are in parentheses.

Given that Se, vitamin C and AT function as antioxidants, it prompted us to examine tissues for oxidative damage. Liver cytosol and plasma protein carbonyl and lipid peroxide concentrations were similar (*P *> 0.05) in guinea pigs fed the different experimental diets (Table 5).

**Table 5:** Protein carbonyl and lipid peroxide concentrations in liver cytosol and plasma of guinea pigs after 12 weeks on the experimental diets^1^

	Protein Carbonyl		Lipid Peroxide	
	
Diet Group	Liver cytosol (μmol/g protein)	Plasma (μmol/g protein)	Liver cytosol (nmol/g wet wt)	Plasma (nmol/mL)
Se-D/MC	3.4 ± 0.1	3.5 ± 0.3	91 ± 13	4.1 ± 0.5
	(n = 9)	(n = 9)	(n = 9)	(n = 7)
Se-M/MC	3.3 ± 0.1	3.1 ± 0.3	87 ± 13	3.9 ± 0.6
	(n = 11)	(n = 11)	(n = 11)	(n = 8)
Se-N/MC	3.4 ± 0.2	3.3 ± 0.2	78 ± 10	2.7 ± 0.6
	(n = 11)	(n = 11)	(n = 11)	(n = 8)
Se-N/NC	3.5 ± 0.2	3.8 ± 0.2	84 ± 8	3.3 ± 0.4
	(n = 11)	(n = 11)	(n = 11)	(n = 8)

^1 ^Data from guinea pigs killed at both 24 and 48 hrs post AA dosing.

^2 ^Values within each column were not significantly different at *P *< 0.05. Values are means ± SEM.

^3 ^n values are in parentheses.

## Discussion

The primary objective of this study was to investigate the sparing effects of dietary Se and AA on tissue vitamin C and AT concentrations. Guinea pigs were chosen for these experiments as they are similar to humans in their inability to make vitamin C and therefore likely provide a more relevant model system compared to previously used cell culture systems [17,18,20] or animal models that have the ability to make vitamin C [7]. Further, we chose to investigate the effects of Se under conditions of marginal AA intake, given that Se may play a more biologically significant role in sparing vitamin C and AT when intake of AA is low.

Only guinea pigs fed the Se-D/MC diet developed paralysis of their limbs. In some cases, the paralysis was severe enough that the guinea pigs died or had to be euthanised. These results are consistent with previous studies demonstrating sensitivity of guinea pigs to disturbances in antioxidant status. Particularly, Se deficiency combined with vitamin E or C deficiency has been reported to cause skeletal muscle damage [24,35]. Further, vitamin E combined with vitamin C deficiency has been shown to promote limb paralysis and death due to severe damage in the brainstem and spinal cord [36].

As part of the study design, guinea pigs were killed after 5 and 12 weeks on the experimental diets at 24 and 48 hrs following AA dosing. Although analyses of vitamin C data at each of the four separate time points revealed little difference between guinea pigs fed different levels of Se, a discernable decreasing trend for vitamin C concentrations in tissues with decreasing dietary Se was observed for week 5, 48 hrs and week 12, 24 hrs guinea pigs. In contrast, no trend was observed for week 5, 24 hrs and week 12, 48 hrs guinea pigs. The reason for the observed Se effect at different times post AA dosing for week 5 and 12 guinea pigs may be explained by differences in the metabolism of the dosed AA between younger (week 5) and older (week 12) guinea pigs. Notably, vitamin C concentrations were higher for guinea pigs killed at 24 compared to 48 hrs post dosing for both week 5 and 12 guinea pigs (data not shown) indicating that vitamin C concentrations rise in tissues following dosing and then fall over time as the vitamin is consumed. Increases in tissue vitamin C concentrations at early times post dosing and low concentrations after an extended time post dosing may mask any effects of Se on vitamin C concentrations. Therefore, if the younger and older guinea pigs metabolised the dosed AA differently (e.g. differences in AA absorption or rate of AA consumption by tissues), it would not be surprising that the Se effects on vitamin C are observed at different times post dosing for week 5 and 12 guinea pigs. However, additional studies are required to definitively show age related differences in AA metabolism in guinea pigs. Nonetheless, whatever the underlying mechanism for this difference, we clearly demonstrate here that dietary Se influences tissue vitamin C concentrations.

In vivo, AA is oxidised to DHAA. We show that Se or AA restriction decreases both the reduced (AA) and oxidised (DHAA) forms of vitamin C. Interestingly, liver was the only tissue that showed an increasing trend in DHAA with decreasing Se in the diet. Impaired regeneration of AA from DHAA with Se restriction may have resulted in accumulation of DHAA in liver, perhaps due to slower elimination of DHAA in liver compared to other tissues.

The observed sparing effects of Se on vitamin C may be explained by Se's role as a component of selenoproteins. It has been reported that the Se-dependent enzyme thioredoxin reductase (TR) can regenerate AA from DHAA [7] and the ascorbyl free radical [8]. Although we were unsuccessful in developing an assay to measure TR activity in guinea pig tissues, it is possible that the low Se diets reduced TR activity which may have contributed to lower concentrations of vitamin C. Decreased antioxidant activity due to decreased activity of Se-dependent enzymes may also have contributed to the lower vitamin C and AT concentrations in tissues, since demand for their antioxidant activity may have been increased. The observed sparing effects of Se on AT may also be partly explained by a secondary effect of Se on AT given that vitamin C may play a role in the regeneration of vitamin E [37,38]. In this regard, marginal AA intake reduced AT concentrations in liver, kidney and spleen.

A reduction in AT with decreased Se or AA intake was only observed in week 12 guinea pigs suggesting that longer-term Se or AA deficiency is more detrimental to tissue AT status than short-term deficiency. Previous studies with guinea pigs failed to observe reductions in AT in tissues with Se [24] or vitamin C [39] deficiency, including liver, which was depleted in AT in this study. However, in contrast to these previous studies, this study was of longer duration and Se-deficient guinea pigs were also fed a marginal AA diet.

AT concentrations were lower in tissues of Se-D/MC compared to Se-M/MC guinea pigs, but not Se-N/MC guinea pigs. Given the absence of significant differences between guinea pigs fed the Se-M/MC or Se-N/MC diets, these data are likely explained by the large variability in tissue AT concentrations between individual guinea pigs. However, these data suggest that marginal amounts of Se are sufficient to maintain tissue AT concentrations.

In most tissues, a large proportion of the total vitamin C was detected in the oxidised form. The large DHAA/AA ratios reported here are consistent with data from an earlier study by Hidiroglou et al [40] that reported comparably large DHAA/AA ratios in tissues of guinea pigs dosed with 1 mg AA/day. In addition, a study by Martensson et al [41] that used different methodology to measure vitamin C detected most of the total vitamin C in liver, lung, kidney and brain of control guinea pigs fed a standard guinea pig chow (Purina) diet as AA; however, when guinea pigs were fed an ascorbate-deficient diet for 21 days, 46 and 45% of the total vitamin C was detected as DHAA in liver and kidney, respectively. It should be noted that liver and kidney vitamin C concentrations reported in this study and that of Hidiroglou et al [40] are comparable to those of the ascorbate-deficient guinea pigs in the study by Martensson et al [41] showing large DHAA/AA ratios in tissues. The low tissue vitamin C concentrations reported in this study reflect the relatively low amounts of AA administered to the guinea pigs. Given these data, it is conceivable that reduced vitamin C intakes and consequently tissue vitamin C concentrations promote an increase in the DHAA/AA ratio in guinea pig tissues.

Se-GSHPx activity decreases with a reduction in Se status and is often used as a measure of Se nutriture in experimental animals, including guinea pigs [24,42,43]. Interestingly, guinea pigs dosed with marginal AA had lower Se-GSHPx activity compared to guinea pigs dosed with normal AA demonstrating a sparing effect of AA on Se-GSHPx activity. It remains to be determined whether the decrease in Se-GSHPx activity reflects a decrease in Se status or change in some other metabolic process that influences Se-GSHPx activity.

Lastly, since decreased antioxidant status can lead to oxidation of cellular components, we examined liver and plasma for oxidative modifications of proteins and lipids. We failed to detect any differences in protein carbonyl and lipid peroxide concentrations in liver cytosols or plasma between guinea pigs fed the different diets. Although these data indicate the absence of severe oxidative modifications to proteins and lipids in these tissues, we cannot rule out the presence of subtle changes that may be detected with more sensitive assays or differences in other markers of oxidative stress.

## Conclusion

In this study, we performed a comprehensive analysis of the sparing effects of Se and AA on vitamin C and AT in guinea pigs, an animal model that is similar to humans and cannot synthesise vitamin C. Dietary restriction of Se and AA decreased both the reduced and oxidised forms of vitamin C as well as AT in tissues. Given these findings and recent studies indicating inadequate Se intakes in certain population groups [44-46], further studies evaluating the health implications and biological significance of reduced vitamin C and E status attributed to a low Se or AA diet are warranted.

## Competing interests

The author(s) declare that they have no competing interests.

## Authors' contributions

JB analysed and interpreted the data and wrote the manuscript. NH and RM performed the vitamin C and AT analyses. RP and PJ measured the plasma GSH. KC and GB performed the protein carbonyl and lipid peroxide determinations. KT measured the liver Se-GSHPx activity. AG assisted in the animal phase of the experiment. MI, SH and NG performed the statistical analyses. ML conceived and coordinated the study and was involved in interpreting the data. All authors read and approved the final manuscript.
